# Exposure to poor hygiene and early life infections and the risk of wheeze or asthma in Latin American children: a systematic review

**DOI:** 10.1186/1939-4551-8-S1-A110

**Published:** 2015-04-08

**Authors:** Cristina Ardura-Garcia, Philip J Cooper, Paul Garner

**Affiliations:** 1 Liverpool School of Tropical Medicine, UK; 2 Laboratorio De Investigaciones Fepis, Ecuador; 3 Pontificia Universidad Catolica Del Ecuador, Ecaudor; 4 St George’s University of London, UK; 5 Liverpool School of Tropical Medicine, UK

## Background

The asthma epidemic in industrialised countries has been explained by the ‘hygiene hypothesis’, according to which early life infections protect against allergic diseases. However, current high asthma rates in Latin American cities seem to be associated with poor hygienic conditions, overcrowding and infections. The aim of the review was to summarise the role of poor hygiene exposures and early life infections on the risk of developing wheeze/asthma amongst Latin American children.

## Methods

MEDLINE, EMBASE, LILACS and CINAHL electronic databases were searched following a pre-defined strategy, with no language, time or publication status restrictions. Observational studies evaluating the association between poor hygiene exposures or infections and asthma/wheeze amongst 4-16 year old Latin American children were included.

## Results

Thirty-two studies met our inclusion criteria: 4 complex studies (2 prospective cohorts), 22 cross-sectional and 6 case-control studies, undertaken between 1987 and 2009. They were mainly urban based and 18 of them were Brazilian studies. Most cross-sectional studies were school-based surveys following International Study of Asthma and Allergies in Childhood (ISAAC) guidelines, and included over 3000 children, while the case-control were hospital-based. Exposures analysed varied greatly. The most frequent outcome used was current wheeze for complex and cross-sectional studies, and asthma (diagnosed by physician) for case-control studies. Methodological quality of studies was acceptable, though 8 studies (excluding case-control) did not carry out any randomization. One quarter of the studies did not clearly describe number of exposures measured and 40% reported on less than 50% of the exposures measured. One third of the reports did not adjust results for possible confounders nor stratified by effect modifiers. The high heterogeneity of study outcomes and exposures studied, precluded conducting a meta-analysis. Great part of the exposures studied showed a non- statistically significant effect on wheeze or asthma risk, with a predominant increased risk after pet contact and acute respiratory infections in early life. Contradictory results between studies were frequent.

## Conclusions

Reporting bias is a common feature in observational studies exploring the association between environmental exposures and risk of wheeze/asthma. The results were highly heterogeneous, possibly be due to difference in asthma phenotypes (atopic vs. non-atopic). Current research seems to indicate a higher risk of wheeze/asthma in Latin America due to pet contact and acute respiratory infections in early life. Large prospective cohort studies are needed in Latin America to clarify the role of hygiene related exposures and early life infections in the development of childhood wheeze or asthma.

